# Synthesis and Biological Activities of Some New (N^α^-Dinicotinoyl)-bis-l-Leucyl Linear and Macrocyclic Peptides

**DOI:** 10.3390/molecules190810698

**Published:** 2014-07-24

**Authors:** Suzan Khayyat, Abd El-Galil Amr

**Affiliations:** 1 Chemistry Department, Faculty of Science for Girls, King Abdulaziz University, Jeddah 31534, Kingdom of Saudi Arabia; 2 Pharmaceutical Chemistry Department, College of Pharmacy, King Saud University, Riyadh 11451, Kingdom of Saudi Arabia, E-Mail: aeamr1963@yahoo.com; 3 Applied Organic Chemistry Department, National Research Center, Dokki 12622, Cairo, Egypt

**Keywords:** 3,5-pyridinedicarboxlic acid, peptide coupling methods, linear and macrocylic peptides, biological activities

## Abstract

A series of linear and macrocyclic peptides **3**–**12** were synthesized using 3,5-pyridinedicarboxylic acid (**1**) as starting material and screened for their antimicrobial, anti-inflammatory and anticancer activities. Bis-ester **3** was prepared from **1** and l-leucine methyl ester. Hydrazinolysis and hydrolysis of dipeptide methyl ester **3** with hydrazine hydrate or 1 N sodium hydroxide afforded compounds **4** and **5**, respectively. Cyclization of the dipeptide **5** with l-lysine methyl ester afforded cyclic pentapeptide ester **6**. Compounds **7**–**9** were synthesized by reacting hydrazide **4** with phthalic anhydride, 1,8-naphthalene anhydride or acetophenone derivatives. Treatment of acid hydrazide **4** with aromatic aldehydes or tetraacid dianhydrides afforded the corresponding bis-dipeptide hydrazones **10a**–**e** and macrocyclic peptides **11** and **12**, respectively. The structures of newly synthesized compounds were confirmed by IR, ^1^H-NMR, MS spectral data and elemental analysis. The detailed synthesis, spectroscopic data, biological and pharmacological activities of the synthesized compounds was reported.

## 1. Introduction

Macrocyclic azacrowns and their complexing derivatives are interesting compounds in the macrocyclic chemistry area [1,2,3]. Peptides rarely function well as drugs due to their low bioavailability and rapid degradation within cells [4]. The conversion of these active peptides into peptidomimetics has been a successful approach for making new biologically active compounds [5]. In addition, the synthesis of some new macrocyclic compounds from pyridinedicarboxylic acid with selected amino acids and screening of their biological activities were reported [6,7,8,9,10,11]. On the other hand, the synthesis of chemosensors is an interesting approach providing accurate analytical tools in different analytical fields. In particular, 2,6-peptido-pyridines exhibit a general ionophoric potency [12] and were used for inventing novel thiocyanate-selective membrane sensors [13]. Recently, some of new heterocyclic and peptide derivatives have been synthesized [14,15] and screened for analgesic and anticonvulsant [16], anticancer [17], and antimicrobial activities [18,19]. Moreover, the cyclo-dodecadepsipetide antibiotic and antitumor valinomycin (from *Streptomyce fulvissimus*, C_54_H_90_N_6_O_18_, CAS: 2001-95-8, NSC: 630175) which is a 36-membered ring, of carboxylic acids and amino acids, is known as a potassium ion specific ionophore. The high affinity for potassium confers this compound its activity as an antibiotic, insecticide, and nematodicide, and particularly in assembling K‏ specific electrodes [20,21,22]. In a similar context, Talma *et al.* [23] reported the synthesis of chiral-bridged bis-coupled amino acid dihydropyidines starting from 3,5-pyridinedicarboxylic acid (dinicotinic acid, 1). These enniatin mimics were also valuable for their chemical activity comparable to enzyme cofactors. Synthesis of some new potential bis-intercallators based on chiral pyridine-2,6-dicarboxamides was equally reported [24]. In view of these observations and as continuation of our previous work [6,7,8,9,10,11,12,13,14,15,16,17,18,19] in macrocyclic and heterocyclic chemistry, we have synthesized some new linear and macrocyclic peptides containing amino acid and pyridine moieties and some of the synthesized compounds were screened for their antimicrobial, anti-inflammatory and anticancer activities compared to the reference drugs.

## 2. Results and Discussion

### 2.1. Chemistry

**Scheme 1:**
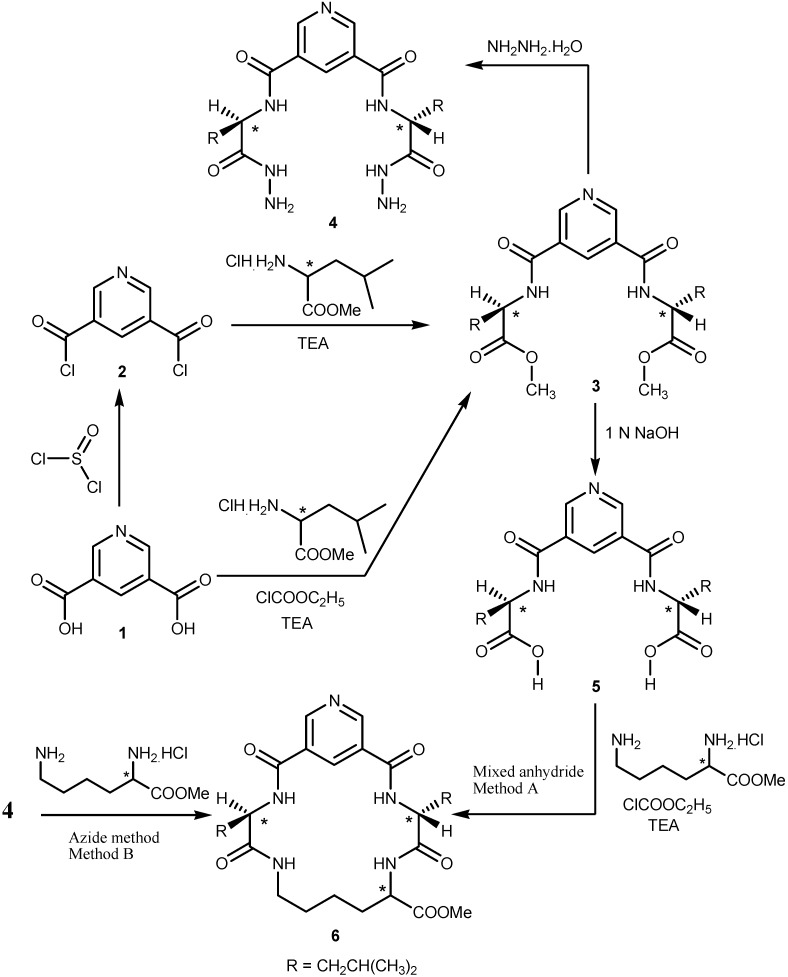
Synthetic route for compounds **3**–**6**.

The synthesis of *N*^α^-dinicotinoyl-bis-(amino acid) methyl ester **3** was based on 3,5-pyridine- dicarbonyl dichloride (**2**) which was obtained by conversion of compound **1** via reaction with thionyl chloride [25]. This acid chloride was then coupled, at low temperature, with l-leucine methyl ester in the presence of triethylamine as organic base. Bis-ester **3** was also prepared from **1** and l-leucine methyl ester in the presence of ethyl chloroformate. Hydrazinolysis of dipeptide methyl ester **3** with hydrazine hydrate in methanol afforded *N*^α^-dinicotinoyl-bis-amino acid hydrazide **4**. Also, hydrolysis of dipeptide methyl ester **3** with 1 N sodium hydroxide in methanol afforded the corresponding *N*^α^-dinicotinoyl-bis-amino acid derivative **5**. Cyclization of the dipeptide **5** with l-lysine methyl ester by the mixed anhydride and azide methods afforded the corresponding cyclic pentapeptide esters **6** (Scheme 1). The IR spectra of **3** confirmed the presence of an aromatic ring, aliphatic hydrogens and an amide linkage in addition to the ester group. The amide linkage was confirmed by its three characteristic IR bands in the *ν* = 1,657, 1,532 and 1,353 cm^−1^ regions (amide I, II and III, respectively). The presence of the ester group is supported by a *ν*(C=O) band in the 1752 cm^−1^ region. In addition, an band was observed at 3,370 cm^−1^, attributed to a hydrogen bonded amide *ν*(NH) absorption. The IR spectra of **4** and **5** also showed the absence of *ν*(C=O, ester), and instead the presence of a broad band at 3,400–3,210 cm^−1^ for *ν*(NHNH_2_, hydrazide) and a band at 1732 cm^−1^ for *ν*(C=O, acid), respectively. The IR and ^1^H-NMR spectra of **6** supported the presence of the ester group by the observation of a *ν* (C=O) band in the 1747 cm^−1^ region and the presence of a singlet (3H) at *δ* = 3.64 ppm for (ester-CH_3_).

**Scheme 2:**
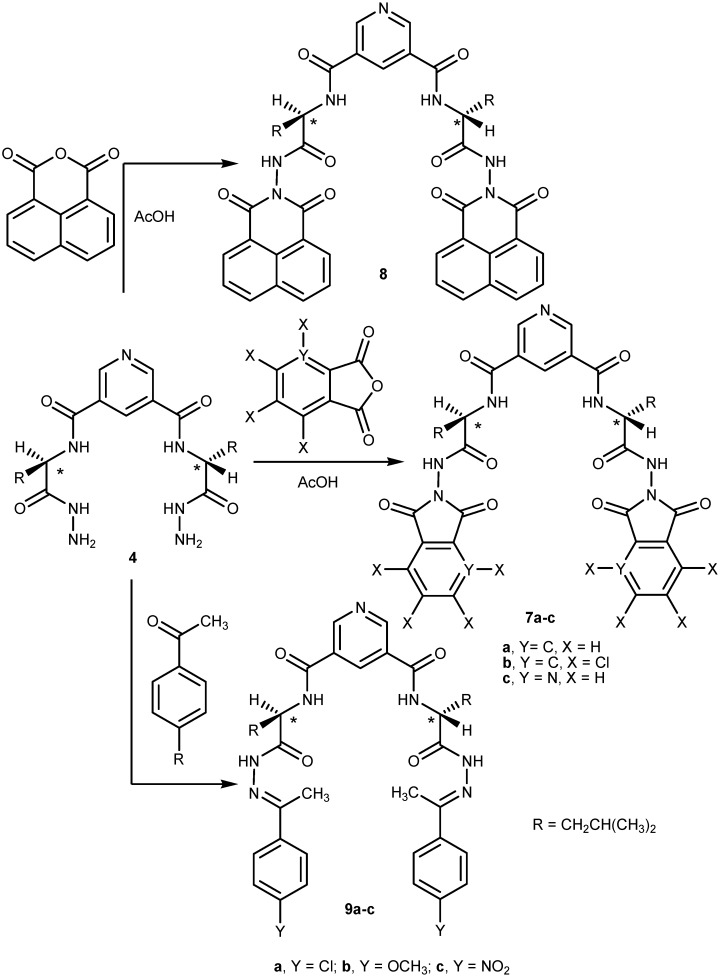
Synthetic route for compounds **7**–**9**.

Compounds **7** and **9** are newly proposed and synthesized candidates obtained via simple condensation of the hydrazide **4** with phthalic anhydride derivatives or 1,8-naphthalene anhydride thus affording the 3,5-bis-imide pyridine derivatives **7a**–**c** and **8**, respectively. Treatment of hydrazide **4** with acetophenone derivatives in acetic acid gave the corresponding hydrazone derivatives **9a**–**c**, respectively (Scheme 2).

**Scheme 3:**
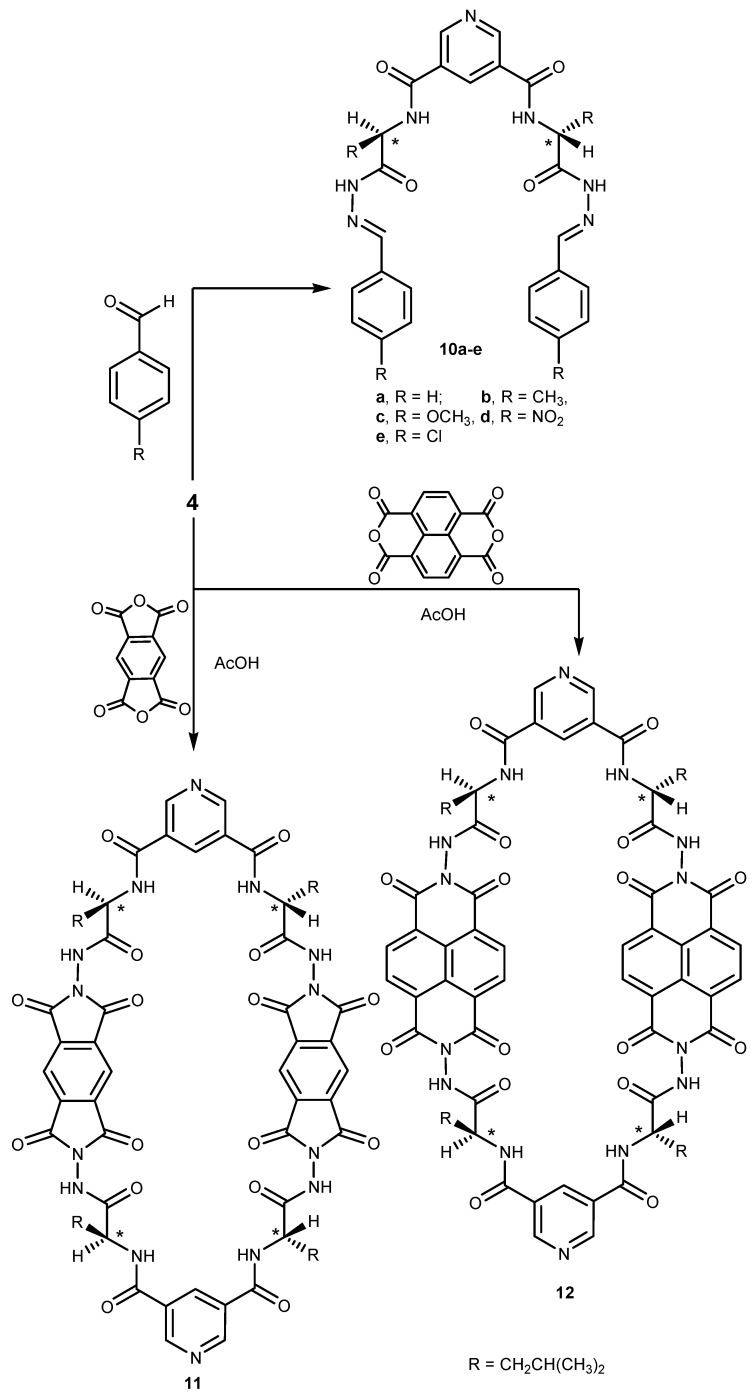
Synthetic route for compounds **10**–**12**.

Finally, treatment of acid hydrazide **4** with aromatic aldehydes in refluxing methanol afforded the corresponding bis-dipeptide hydrazones **10a**–**e**. In addition, condensation of the same hydrazide **4** with selected tetraacid dianhydrides, namely, 1,2,4,5-benzenetetracarboxylic dianhydride or 1,8,4,5-naphthalenetetracarboxylic dianhydride in refluxing acetic acid afforded the corresponding macrocyclic octaaamide-tetraimide pyridine derivatives **11** and **12**, respectively (Scheme 3).

### 2.2. Pharmacological Screening

#### 2.2.1. Antimicrobial Activity

The newly synthesized compounds **3**–**12** were tested for their preliminary antimicrobial activity against different microorganisms representing Gram-positive (*Bacillus subtilis*, *Bacillus aureus* and *Staphylococcus aureus*), Gram-negative bacteria (*Escherichia coli*), yeast (*Candida albican*) and fungi (*Aspergillus niger*). The obtained results are summarized in Table 1.

**Table 1:** Antimicrobial activities of some newly synthesized compounds.

Compound No.	Inhibition Zome (cm)					
	Gram^+^ ve			Gram^−^ ve	Yeast	Fungi
	*Bacillus subtilis*	*Bacillus aureus*	*Staphylococcus aureus*	*Escherichia coli*	*Candida albican*s	*Aspergillus niger*
**3**	1.46	1.65	1.76	0.62	-	1.78
**4**	1.85	1.92	1.80	0.80	0.92	1.56
**5**	1.68	1.14	1.76	0.64	-	1.72
**6**	1.82	1.50	1.54	0.65	-	1.75
**7a**	1.55	1.84	1.58	0.75	1.02	1.64
**7b**	1.46	1.80	1.45	0.66	-	1.95
**7c**	1.72	1.56	1.76	0.74	0.95	1.88
**8**	1.90	1.94	1.95	0.90	0.98	2.00
**9a**	1.84	1.88	1.76	0.92	1.10	2.01
**9b**	1.60	1.74	1.72	0.75	1.00	1.88
**9c**	1.72	1.22	1.66	0.60	-	1.76
**10a**	1.56	1.45	1.56	0.66	-	1.68
**10b**	1.80	1.95	1.85	0.78	0.95	1.56
**10c**	1.76	1.56	1.64	0.80	1.00	1.95
**10d**	1.85	2.00	1.92	0.90	0.96	2.00
**10e**	1.65	1.96	1.80	0.78	0.94	1.48
**11**	1.60	1.17	1.98	0.64	-	1.75
**12**	1.65	1.83	1.65	0.64	-	1.58
**Chloramphenicol**	2.00	2.10	2.00	0.95	-	-
**Fusidic Acid**	-	-	-	-	1.9	1.9

#### 2.2.2. Anti-Inflammatory Activity

All animals were obtained from the Animal House Colony, Research Institute of Ophthalmology, Giza, Egypt. The newly synthesized compounds were screened for their anti-inflammatory activities using male albino rats (Table 2).

##### Purpose and Rational

For the determination of the antiphlogistic potency of the synthesized compounds, two standard tests were realized at a dose level 2.5 and 5 mg/kg body weight of the rats, namely the protection against carrageenan-induced edema according to Winter *et al.* [26] and the inhibition of plasma PGE2. The latter is known as a good confirming indicator for carrageenan-induced rat paw edema [27]. Regarding the protection against carrageenan-induced edema, all tested compounds were found to be more potent than diclofenac potassium. For these compounds, a similar activity profile was realized for the inhibition of plasma PGE2. Regarding the descending order of the anti-inflammatory activities at 5 mg/kg is **6**, **12**, **10a**, **7a**, **4**, **3**, **5**, **10d**, **7c**, **9a**, **9b** and **8**, with compounds **6**, **12**, **10a**, **7a** and **4** are the most active products.

**Table 2:** Anti-inflammatory activities of some new synthesized compounds.

Compound No.	Dose mg/kg	% Protection against Edema	% Inhibition of Plasma PGE2
**3**	2.5	86.16 ± 0.052	59.45 ± 0.050
	5.0	98.18 ± 0.053	81.10 ± 0.056
**4**	2.5	93.45 ± 0.074	75.66 ± 0.040
	5.0	98.56 ± 0.060	80.01 ± 0.058
**5**	2.5	85.14 ± 0.056	61.16 ± 0.041
	5.0	96.18 ± 0.067	77.14 ± 0.036
**6**	2.5	92.14 ± 0.076	84.18 ± 0.052
	5.0	99.08 ± 0.062	86.01 ± 0.030
**7a**	2.5	97.45 ± 0.054	81.30 ± 0.044
	5.0	98.86 ± 0.046	82.48 ± 0.052
**7c**	2.5	91.12 ± 0.064	73.48 ± 0.049
	5.0	95.14 ± 0.075	78.55 ± 0.035
**8**	2.5	78.88 ± 0.090	57.57 ± 0.045
	5.0	92.00 ± 0.060	76.00 ± 0.035
**9a**	2.5	82.16 ± 0.076	58.96 ± 0.024
	5.0	93.16 ± 0.064	74.58 ± 0.040
**9b**	2.5	79.34 ± 0.081	57.90 ± 0.040
	5.0	92.92 ± 0.090	77.14 ± 0.052
**10a**	2.5	90.32 ± 0.035	62.12 ± 0.051
	5.0	99.10 ± 0.036	83.01 ± 0.049
**10d**	2.5	81.00 ± 0.072	63.14 ± 0.023
	5.0	95.82 ± 0.062	79.88 ± 0.031
**12**	2.5	90.48 ± 0.090	63.16 ± 0.036
	5.0	99.36 ± 0.080	86.18 ± 0.071
**Diclofenac Potassium**	2.5	70.14 ± 0.064	54.00 ± 0.041
	5.0	75.23 ± 0.083	70.00 ± 0.051

All results were significantly different from the standard and normal control value at *p* ≤ 0.05.

### 2.3. Anticancer Activity

#### *In Vitro* Anti-Breast Cancer Activities

Compounds **3**, **4**, **5**, **6**, **7a**, **7c**, **9a**, **9b**, **10a**, **10d**, **12** showed *in vitro* activity against the MCF7, MDAMB231, HS578T and MDAMB435 breast cancer cell lines (Table 3). The most potent compounds were against the MCF7 cell line, so we resorted to the xenograft model of MCF7 which revealed that these compounds were also active *in vivo* (Table 4). 

**Table 3:** *In vitro* anti-tumor activity of the tested compounds was evaluated in different cell lines of breast cancer.

Compound No.	IC_50_ (μM ) for Tested Compounds against Breast Cell Lines						
	MCF7	MDAMB231	HS578T	MDAMB435	MDN	BT549	T47D
**3**	0.00029	3.23	3.34	56.38	0.00	0.00	0.00
**4**	0.00026	2.76	2.78	45.47	0.00	0.00	0.00
**5**	0.00017	2.34	2.56	33.66	0.00	0.00	0.00
**6**	0.00075	4.56	8.89	88.76	0.00	0.00	0.00
**7a**	0.00086	7.89	9.75	89.87	0.00	0.00	0.00
**7c**	0.00099	9.65	14.45	92.99	0.00	0.00	0.00
**9a**	0.00097	8.65	11.43	90.98	0.00	0.00	0.00
**9b**	0.00064	3.56	7.47	78.65	0.00	0.00	0.00
**10a**	0.00014	1.23	1.54	10.55	0.00	0.00	0.00
**10d**	0.00035	3.67	5.67	57.47	0.00	0.00	0.00
**12**	0.00056	3.56	6.65	68.56	0.00	0.00	0.00

**Table 4:** *In vivo* anti-tumor activity of the tested compounds was evaluated in an MCF-7 mouse xenograft model of breast cancer.

Compound No.	Tumor Growth Vt/Vo for Compounds after Times in Days										
	0	2	4	6	8	10	12	14	16	18	20
**Control**	1.00	1.57	2.11	4.87	9.67	12.55	24.34	26.45	28.30	39.90	41.12
**3**	1.00	1.40	1.56	1.87	2.01	2.89	3.54	5.10	6.12	7.14	8.12
**4**	1.00	1.34	1.45	1.77	1.91	2.77	3.44	4.89	5.56	6.12	7.14
**5**	1.00	1.21	1.28	1.65	1.81	2.67	3.21	4.45	5.22	5.77	6.23
**6**	1.00	1.40	1.67	2.45	2.79	3.56	4.57	5.98	7.01	8.88	9.56
**7a**	1.00	1.41	1.71	2.78	3.20	3.78	4.78	6.11	7.16	8.90	9.77
**7c**	1.00	1.42	1.72	2.98	3.40	4.00	5.01	6.18	7.28	8.99	9.99
**9a**	1.00	1.41	1.70	2.89	3.30	3.90	4.90	6.13	7.13	8.79	9.88
**9b**	1.00	1.42	1.78	2.34	2.67	3.45	4.56	5.90	6.88	8.89	9.45
**10a**	1.00	1.14	1.24	1.56	1.76	2.54	3.11	4.32	5.12	5.89	6.12
**10d**	1.00	1.40	1.6	1.99	2.34	3.23	4.34	5.55	6.66	8.45	9.00
**12**	1.00	1.41	1.65	2.14	2.56	3.25	4.39	5.78	6.79	8.68	9.14

## 3. Experimental Section

### 3.1. General Information

Melting points were determined in open glass capillary tubes with an Electro Thermal Digital melting point apparatus IA9100 (Shimadzu, Tokyo, Japan) and are uncorrected. Elemental microanalysis for carbon, hydrogen and nitrogen (Microanalytical Unit, NRC) was found within the acceptable limits of the calculated values (±0.1%). Infrared spectra (KBr) were recorded on a Nexus 670 FTIR Fourier Transform infrared spectrometer (Nicolet, Norwalk, CT, USA). Proton and carbon nuclear magnetic resonance (^1^H-NMR—500 MHz and ^13^C-NMR—125 MHz) spectra were run in DMSO-*d_6_* on a JEOL 500 MHz instrument (Tokyo, Japan). Mass spectra were run on a MAT Finnigan SSQ 7000 spectrometer (Madison, WI, USA), using the electron impact technique (EI). Analytical thin layer chromatography (TLC) was performed on silica gel aluminum sheets, 60F_254_ (E. Merck, Darmstadt, Germany). Specific optical rotations were measured with an Optronic, P8000 polarimeter (A. Krauss, Hamburg, Germany) in a 1 dm length observation tube, at the indicated conditions, and according to the equation:

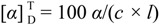
(1)
where: *α* = observed rotation angle, D = sodium line (*λ* = 589 nm), *c* = concentration (g/100 mL), *l* = path length in dm and *T* = experimental temperature (°C).

*3,5-Bis-(Methyl l-Leucenylcarbonyl)pyridine carboxylate* (**3**). Method (A): To a stirred cold mixture (−15 °C) of 3,5-pyridinedicarboxylic acid (**1**, 0.167 g, 1 mmol) in cold dry dichloromethane (100 mL) and ethyl chloroformate (0.216 g, 2 mmol), triethylamine (0.202 g, 2 mmol) was added, then after 10 min, l-leucine methyl ester (2 mmol) was added then stirred at −15 °C for 3 h then for 12 h at r.t. The reaction mixture was washed with water, 1 N hydrochloric acid, 1 N sodium bicarbonate and finally with water and dried over anhydrous calcium chloride. Solvent was evaporated under reduced pressure to dryness and the obtained residue was triturated with *n*-hexane to give the corresponding 3,5-bis methyl ester pyridine derivative **3**. Method (B): To a solution of l-leucine methyl ester (2 mmol) in dichloromethane (15 mL), pyridine-3,5-dicarbonyl dichloride (**2**, 0.204 g, 1 mmol) was added at −10 °C with stirring. Triethylamine (0.2 mL, 2 mmol) was added dropwise to the reaction mixture with stirring in order to keep the reaction mixture slightly basic (pH ≈ 8). Stirring was maintained for 3 h at (−15 °C) and 12 h at r.t. The reaction mixture was then washed with water, 1 N hydrochloric acid, 1 N sodium bicarbonate and finally with water and dried over anhydrous calcium chloride. Solvent was evaporated under reduced pressure to dryness to give the corresponding 3,5-bismethyl ester **3** as identified by melting point and TLC in comparison with authentic sample prepared according to method A. Yield: 65% [A], 92 [B]; m.p. 118–120 °C; 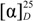:−132.5 (c = 0.5, DMF). IR (KBr): *ν* = 3370 (NH str.), 3045 (CH-Ar), 29525 (CH-aliph.), 1752 (C=O, ester), 1657, 1532, 1253 (C=O, amide I, II and III) cm^−1^. ^1^H-NMR: *δ* = 0.95–0.88 (d, 12H, 4CH_3_), 1.80–1.73 (m, 6H, 2CH_2_ + 2CH), 3.81 (s, 6H, 2OCH_3_), 4.84 (t, 2H, 2CHNH), 8.48 (s, 2H, 2NH, exchangeable with D_2_O), 8.40, 9.01 (2s, 3H, Pyr-H). ^13^C-NMR: *δ* = 22.35 (2C, 2CH), 22.82 (4C, 4CH3), 41.04 (2C, 2CH2), 49.56 (2C, 2CH), 52.08 (2C, 2CH3), 131.56, 140.03, 152.10 (5C, Pyr-C), 167.45 (2C, 2C=O), 172.08 (2C, 2C=O). MS (EI, 70 eV): *m/z* (%) = 421 [M^+^, 22], 278 (32), 247 (100), 144 (16). C_21_H_31_N_3_O_6_ (421.49): calcd. C 59.84, H 7.41, N 9.97; found C 59.78, H 7.34, N 9.90.

*N^2^,N^2^′-(Pyridine-3,5-dicarbonyl)-di-l-leucyl hydrazide* (**4**). Hydrazine hydrate (0.8 mL, 16 mmol) was added to a methanolic solution (10 mL) of **3** (1 mmol). The reaction mixture was refluxed for 10 h, after which the solvent was evaporated under reduced pressure. The obtained residue was triturated with ether, filtered off, and recrystallized from dioxane to afford the corresponding 3,5-bis hydrazide derivative **4**. Yield: 78%; m.p. 215–217 °C; 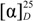: −98.8 (c = 0.5, DMF). IR (KBr): *ν* = 3400–3210 (NH, NH_2_), 3050 (CH-Ar), 2954 (CH-aliph.), 1662, 1530, 1245 (C=O, amide I, II and III) cm^−1^. ^1^H-NMR: *δ* = 0.98–0.86 (m, 12H, 4CH_3_), 1.82–1.73 (m, 6H, 2CH_2_ + 2CH), 3.45 (s, 4H, 2 NH_2_, exchangeable with D_2_O), 4.64 (t, 2H, 2CHNH), 8.58, 8.98 (2s, 4H, 4NH, exchangeable with D_2_O), 8.48, 9.22 (2s, 3H, Pyr-H). ^13^C-NMR: *δ* = 22.84 (4C, 4CH_3_), 22.95 (2C, 2CH), 40.98 (2C, 2CH_2_), 42.86 (2C, 2CH), 132.06, 140.10, 152.25 (5C, Pyr-C), 167.53 (2C, 2C=O), 170.16 (2C, 2C=O). MS (EI, 70 eV): *m/z* (%) = 422 [M^+^+1, 8], 374 (12), 247 (100), 134 (86). C_19_H_31_N_7_O_4_ (421.49): calcd. C 54.14, H 7.41, N 23.26; found C 54.05, H 7.35, N 23.20.

*N,N′-Bis(1-Carboxy-2-l-leucyl)-3,5-(diaminocarbonyl)pyridine* (**5**). Sodium hydroxide (1 N, 25 mL) was added dropwise to a cold and stirred ethanolic solution (1 mmol, −5 °C) of the ester **3**. Stirring was continued at that temperature for 2 h and then for 12 h at room temperature followed by evaporation of the solvent. The cold reaction mixture was acidified with 1 N hydrochloric acid to pH ≈ 3, and the obtained solid was filtered off, washed with cold water, dried, and recrystallized from ethanol to afford the title acid derivative **5**. Yield: 86%; m.p. 168–170 °C; 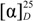: −104.5 (c = 0.5, DMF). IR (KBr): *ν* = 3578–3385 (OH, NH), 3065 (CH-Ar), 2958 (CH-aliph.), 1732 (C=O, acid), 1664, 1536, 1248 (C=O, amide I, II and III) cm^−1^. ^1^H-NMR: *δ* = 1.05–0.96 (m, 12H, 4CH_3_), 1.80–1.71 (m, 6H, 2CH_2_ + 2CH), 4.60 (t, 2H, 2CHNH), 8.68 (s, 2H, 2NH, exchangeable with D_2_O), 8.42, 9.18 (2s, 3H, Pyr-H), 12.24 (s, 2H, 2OH, exchangeable with D_2_O). ^13^C-NMR: *δ* = 22.12 (2C, 2CH), 22.92 (4C, 4CH_3_), 39.95 (2C, 2CH_2_), 52.35 (2C, 2CH), 132.12, 139.88, 152.18 (5C, Pyr-C), 167.42 (2C, 2C=O), 174.56 (2C, 2C=O). MS (EI, 70 eV): *m/z* (%) = 393 [M^+^, 16], 360 (32), 246 (24), 134 (100). C_19_H_27_N_3_O_6_ (393.43): calcd. C 58.00, H 6.92, N 10.68; found C 57.90, H 6.87, N 10.62.

*Synthesis of Tripeptide Derivatives: Cyclo-(Na-dinicotinoyl)-bis-(l-leucyl)-l-lysine methyl ester* (**6**)*.* Mixed anhydride method (A): Ethyl chloroformate (0.2 mL, 2 mmol) was added to a stirred and cold (−15 °C) dichloromethane solution (20 mL) of the corresponding acid **5** (1 mmol), containing TEA (2 mmol). The reaction mixture was stirred for additional 20 min., then a cold (−15 °C) dichloromethane solution (20 mL) of free l-lysine methyl ester (1 mmol) was added. Stirring was maintained for 3 h at −15 °C then for 12 h at room temperature. The reaction mixture was washed with water, 1 N sodium bicarbonate, 1 N potassium hydrogen sulfate and water then dried over anhydrous sodium sulfate. The solvent was evaporated under reduced pressure to dryness and the obtained oily residue was solidified by trituration with dry ether/*n*-hexane mixture. The crude product was purified by preparative thin layer chromatography using (CHCl_3_/MeOH, 9:1) as eluent to give the corresponding cyclic tripeptide methyl ester **6**. Azide method (B): A cold mixture (−15 °C) of dihydrazide derivative **4** (1 mmol) in hydrochloric acid (6N, 2 mL) and glacial acetic acid (1 mL) was stirred for 10 min then an aqueous solution of sodium nitrite (5 M, 2 mL) was added. Stirring was maintained for 30 min. at the same temperature, after which the reaction mixture was extracted with ether (60 mL), washed with cold water, 5% sodium bicarbonate and water then dried over anhydrous sodium sulfate. The cold ethereal azide solution (−15 °C) was added to free l-lysine methyl ester (1 mmol). Stirring was maintained for 5 h at the same temperature then for 20 h at room temperature. The reaction mixture was washed with water, 5% potassium hydrogen sulfate and water then dried over anhydrous sodium sulfate. Ether was evaporated to dryness and the obtained oily residue was solidified by trituration with dry ether/*n-*hexane mixture. The crude product was purified by preparative thin layer chromatography using (CHCl_3_/MeOH, 9:1) as eluent to give the corresponding cyclic tripeptide methyl ester **6** as identified by melting point and TLC in comparison with authentic sample prepared according to method A. Yield: 76% [A], 48% [B]; m.p. 156–158 °C; 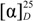: = −178.6 (c = 0.5, DMF). IR (KBr): *ν* = 3410–3335 (NH), 3062 (CH-Ar), 2955 (CH-aliph.), 1747 (C=O, ester), 1660, 1528, 1341 (C=O, amide I, II and III) cm^−1^. ^1^H-NMR: *δ* = 1.05–0.96 (m, 12H, 4CH_3_), 1.34–1.24 (m, 2H, CH_2_), 1.56–1.53 (m, 2H, CH_2_), 1.86–1.75 (m, 6H, 2CH_2_ + 2CH), 2.45–2.36 (m, 2H, CH_2_), 3.30–3.20 (m, 2H, CH_2_), 3.64 (s, 3H, OCH_3_), 4.42 (t, 1H, CHNH), 4.58 (t, 2H, 2CHNH), 8.62, 8.98 (2s, 4H, 4NH, exchangeable with D_2_O), 8.32, 9.15 (2s, 3H, Pyr-H). ^13^C-NMR: *δ* = 18.45, 30.60, 33.85, 41.45 (4C, 4CH_2_), 21.86 (2C, 2CH), 22.34 (4C, 4CH_3_), 40.92 (2C, 2CH_2_), 51.45 (2C, 2CH), 51.14 (1C, CH_3_), 53.96 (1C, CH), 131.86, 139.84, 152.00 (5C, Pyr-C), 167.14 (2C, 2C=O), 169.62 (2C, 2C=O), 171.05 (1C, C=O, ester). MS (EI, 70 eV): *m/z* (%) = 518 [M^+^, 18], 486 (45), 403 (52), 246 (34), 188 (100). C_26_H_39_N_5_O_6_ (517.62): calcd. C 60.33, H 7.59, N 13.53; found C 60.26, H 7.52, N 13.45.

*Synthesis of N^α^-Dinicotinoyl-bis-[l-leucyl]-substitutedphthalic-1,2-hydrazine conjugates* **7a**–**c** and **8**. A suspension of **4** (0.34 g, 1 mmol) and acid anhydrides, namely, phthalic, 3,4,5,6-tetrachloro-phthalic, 2,3-pyridinedicarboxylic or 1,8-naphthalenedicarboxylic anhydrides (2 mmol) in glacial acetic acid (50 mL) was refluxed for 3 h. The solvent was evaporated under reduced pressure, the residue was solidified with diethyl ester. The obtained solid was filtered off, dried and crystallized from DMF/water to give the corresponding 3,5-bis-tetracarboxamidodiimide derivatives **7a**–**c** and **8**, respectively.

*2,6-Bis[(N*-[4-methyl-1-(1H-isoindole-1,3(2H)-dion)-pentan]-*3-carboxamido]pyridine* (**7a**). Yield: 82%; m.p. 205–207 °C; 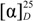: −84.4 (c = 0.5, DMF). IR (KBr): *ν* = 3590–3480 (NH), 3056 (CH-Ar), 2960 (CH-aliph), 1753 (C=O, imide), 1658, 1532, 1322 (C=O, amide I, II and III) cm^−1^. ^1^H-NMR: *δ* = 1.01–0.92 (m, 12H, 4CH_3_), 1.83–1.74 (m, 6H, 2CH_2_ + 2CH), 4.66 (t, 2H, 2CHNH), 7.45–8.10 (m, 8H, Ar-H), 8.62, 9.08 (2s, 4H, 4NH, exchangeable with D_2_O), 8.38, 9.22 (2s, 3H, Pyr-H). ^13^C-NMR: *δ* = 22.56 (4C, 4CH_3_), 22.95 (2C, 2CH), 41.16 (2C, 2CH_2_), 52.86 (2C, 2CH), 131.48, 140.10, 152.12 (5C, Pyr-C), 167.42 (2C, 2C=O), 169.84 (2C, 2C=O), 163.56 (4C, 4C=O), 127.15, 132.02, 132.86 (12C, Ar-C). MS (EI, 70 eV): *m/z* (%) = 682 [M^+^, 14], 520 (21), 274 (86), 247 (53), 189 (100). C_35_H_35_N_7_O_8_ (681.69): calcd. C 61.67, H 5.18, N 14.38; found C 61.60, H 5.12, N 14.33.

*2,6-Bis[(N*-[4-methyl-1-(4,5,6,7-tetrachloro-1H-isoindole-1,3(2H)-dion)-pentan]-*3-carboxamido]-pyridine* (**7b**). Yield: 64%; m.p. 232–234 °C; 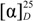: −96.5 (c = 0.5, DMF). IR (KBr): *ν* = 3532–3450 (NH), 3058 (CH-Ar), 2961 (CH-aliph.), 1750 (C=O, imide), 1656, 1528, 1318 (C=O, amide I, II and III) cm^−1^. ^1^H-NMR: *δ* = 1.00–0.90 (m, 12H, 4CH_3_), 1.84–1.76 (m, 4H, 2CH_2_ + 2CH), 4.65 (t, 2H, 2CHNH), 8.60, 9.04 (2s, 4H, 4NH, exchangeable with D_2_O), 8.45, 9.24 (2s, 3H, Pyr-H). ^13^C-NMR: *δ* = 22.62 (4C, 4CH_3_), 22.94 (2C, 2CH), 41.24 (2C, 2CH2), 52.88 (2C, 2CH), 131.44, 140.04, 152.10 (5C, Pyr-C), 167.36 (2C, 2C=O), 169.92 (2C, 2C=O), 164.04 (4C, 4C=O), 128.25, 132.95, 138.36 (12C, Ar-C). MS (EI, 70 eV): *m/z* (%) = 957 [M^+^, 8], 542 (14), 410 (16), 324 (45), 247 (45), 245 (100). C_35_H_27_Cl_8_N_7_O_8_ (957.25): calcd. C 43.91, H 2.84, Cl 29.63, N 10.24; found C 43.86, H 2.75, Cl 29.55, N 10.18.

*2,6-Bis[(N*-[4-methyl-1-(5H-pyrrolo[3,4-b]*pyridine-5,7(6H)-dion)-pentan]-3-carboxamido]pyridine* (**7c**). Yield: 58%; m.p. 187–189 °C; 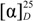: −114.2 (c = 0.5, DMF). IR (KBr): *ν* = 3565–3328 (NH), 3048 (CH-Ar), 2962 (CH-aliph.), 1748 (C=O, imide), 1655, 1536, 1320 (C=O, amide I, II and III) cm^−1^. ^1^H-NMR: *δ* = 1.00–0.94 (m, 12H, 4 CH_3_), 1.85–1.69 (m, 6H, 2 CH_2_ + 2CH), 4.65 (t, 2H, 2CHNH), 8.72, 9.04 (2d, 4H, Pyr-H), 8.02 (t, 2H, Pyr-H), 9.18, 8.64 (2s, 4H, 4NH, exchangeable with D_2_O), 8.36, 9.10 (2s, 3H, Pyr-H). ^13^C-NMR: *δ* = 22.56 (4C, 4CH_3_), 22.92 (2C, 2CH), 41.25 (2C, 2CH_2_), 52.95 (2C, 2CH), 131.46, 140.10, 151.98 (5C, Pyr-C), 167.22 (2C, 2C=O), 170.02 (2C, 2C=O), 164.12, 165.05 (4C, 4C=O), 127.85, 128.15, 138.32, 146.10, 152.45 (10C, Pyr-C). MS (EI, 70 eV): *m/z* (%) = 684 [M^+^, 14], 494 (25), 304 (85), 247 (95), 190 (100). C_33_H_33_N_9_O_8_ (683.67): calcd. C 57.97, H 4.87, N 18.44; found C 57.90, H 4.80, N 18.38.

*2,6-Bis[(N*-[4-methyl-1-(1H-benzo[de]*isoquinoline-1,3(2H)-dion)-pentan]-3-carboxamido]pyridine* (**8**). Yield: 68%; m.p. 198–200 °C; 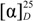: −108.8 (c = 0.5, DMF). IR (KBr): *ν* = 3548–3452 (NH), 3056 (CH-Ar), 2950 (CH-aliph.), 1746 (C=O, imide), 1656, 1532, 1328 (C=O, amide I, II and III) cm^−1^. ^1^H-NMR: *δ* = 1.00–0.95 (m, 12H, 4 CH_3_), 1.85–1.73 (m, 6H, 2 CH_2_ + 2CH), 4.62 (t, 2H, 2CHNH), 7.55–8.05 (m, 12H, Ar-H), 8.68, 9.12 (2s, 4H, 4NH, exchangeable with D_2_O), 8.35, 9.08 (2s, 3H, Pyr-H). ^13^C-NMR: *δ* = 22.55 (4C, 4CH_3_), 22.98 (2C, 2CH), 41.25 (2C, 2CH_2_), 52.85 (2C, 2CH), 131.48, 140.10, 152.15 (5C, Pyr-C), 167.42 (2C, 2C=O), 169.88 (2C, 2C=O), 158.76 (4C, 4C=O), 123.45, 125.02, 128.20, 130.54, 137.05 137.65 (20C, Ar-C). MS (EI, 70 eV): *m/z* (%) = 782 [M^+^, 6], 570 (12), 324 (25), 246 (100), 134 (95). C_43_H_39_N_7_O_8_ (781.81): calcd. C 66.06, H 5.03, N 12.54; found C 66.00, H 4.96, N 12.50.

*Synthesis of N^α^-Dinicotinoyl-bis[l-leucyl]-p-substituted phenylhydrazones* **9a**–**c**. A stirred solution of hydrazide **4** (1 mmol) and active carbonyl derivatives, namely, *p*-chloro-, *p*-methoxy- or *p*-nitroacetophenones (2 mmol) in acetic acid (30 mL) was refluxed for 4 h. The reaction mixture was allowed to cool and the obtained solid product was filtered off, dried, and recrystallized from the proper solvents to give the corresponding hydrazone derivatives **9a**–**c**, respectively.

*2,6-Bis {N-[1-{(2E)-2-[1-(4-chlorophenyl)ethylidene]hydrazinyl}-4-methyl-1-oxopentan]-3-carboxamido}pyridine* (**9a**). Yield: 72%; m.p. 165–167 °C (AcOH/EtOH); 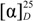: −106.6 (c = 0.5, DMF). IR (KBr): *ν* = 3510–3432 (NH), 3054 (CH-Ar), 2945 (CH-aliph.), 1660, 1526, 1316 (C=O, amide I, II and III) cm^−1^. ^1^H-NMR: *δ* = 0.85 (s, 6H, 2CH_3_), 0.98–0.92 (m, 12H, 4 CH_3_), 1.82–1.72 (m, 6H, 2 CH_2_ + 2CH), 4.64 (t, 2H, 2CHNH), 7.30–7.65 (m, 8H, Ar-H), 9.08, 8.35 (2s, 4H, 4NH, exchangeable with D_2_O), 8.42, 9.16 (2s, 3H, Pyr-H). ^13^C-NMR: *δ* = 14.05 (2C, 2CH_3_), 22.48 (4C, 4CH_3_), 22.95 (2C, 2CH), 41.35 (2C, 2CH_2_), 52.78 (2C, 2CH), 131.56, 140.18, 152.12 (5C, Pyr-C), 167.35 (2C, 2C=O), 172.84 (2C, 2C=O), 168.12 (2C, C=N), 128.45, 129.68, 131.89, 135.65 (12C, Ar-C). MS (EI, 70 eV): *m/z* (%) = 695 [M^+^, 10], 582 (8), 416 (24), 359 (100), 246 (95). C_35_H_41_Cl_2_N_7_O_4_ (694.65): calcd. C 60.52, H 5.95, Cl 10.21, N 14.11; found C 60.45, H 5.90, Cl 10.15, N 14.05.

*2,6-Bis {N-[1-{(2E)-2-[1-(4-methoxyphenyl)ethylidene]hydrazinyl}-4-methyl-1-oxopentan]-3-carboxamido}pyridine* (**9b**). Yield: 64%; m.p. 145–47 °C (DMF/H_2_O); 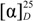: −112.5 (c = 0.5, DMF). IR (KBr): *ν* = 3498–3386 (NH), 3065 (CH-Ar), 2943 (CH-aliph.), 1658, 1530, 1337 (C=O, amide I, II and III) cm^−1^. ^1^H-NMR: *δ* = 0.86 (s, 6H, 2CH_3_), 0.99–0.94 (m, 12H, 4 CH_3_), 1.80–1.74 (m, 6H, 2 CH_2_ + 2CH), 3.65 (s. 6H, 2 OCH_3_), 4.62 (t, 2H, 2CHNH), 7.02–7.60 (m, 8H, Ar-H), 7.85, 8.45 (2s, 4H, 4NH, exchangeable with D_2_O), 8.36, 9.12 (2s, 3H, Pyr-H). ^13^C-NMR: *δ* = 13.95 (2C, 2CH_3_), 22.56 (4C, 4CH_3_), 23.05 (2C, 2CH), 41.36 (2C, 2CH_2_), 52.84 (2C, 2CH), 56.04 (2C, 2OCH_3_), 131.58, 140.10, 152.10 (5C, Pyr-C), 167.28 (2C, 2C=O), 172.92 (2C, 2C=O), 168.15 (2C, C=N), 114.02, 125.65, 129.85, 162.15 (12C, Ar-C). MS (EI, 70 eV): *m/z* (%) = 686 [M^+^, 5], 522 (6), 276 (24), 246 (100). C_37_H_47_N_7_O_6_ (685.81): calcd. C 64.80, H 6.91, N 14.30; found C 64.72, H 6.83, N 14.24.

*2,6-Bis {N-[1-{(2E)-2-[1-(4-nitrophenyl)ethylidene]hydrazinyl}-4-methyl-1-oxopentan]-3-carboxamido}pyridine* (**9c**). Yield: 56%; m.p. 235–237 °C (AcOH/H_2_O); 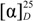: −122.4 (c = 0.5, DMF). IR (KBr): *ν* = 3495–3435 (NH), 3050 (CH-Ar), 2945 (CH-aliph.), 1655, 1531, 1324 (C=O, amide I, II and III) cm^−1^. ^1^H-NMR: *δ* = 0.88 (s, 6H, 2CH_3_), 1.00–0.96 (m, 12H, 4 CH_3_), 1.85–1.64 (m, 6H, 2 CH_2_ + 2CH), 4.65 (t, 2H, 2CHNH), 7.65–8.05 (m, 8H, Ar-H), 9.10, 8.36 (2s, 4H, 4NH, exchangeable with D_2_O), 8.38, 9.10 (2s, 3H, Pyr-H). ^13^C-NMR: *δ* = 13.98 (2C, 2CH_3_), 22.54 (4C, 4CH_3_), 22.98 (2C, 2CH), 41.32 (2C, 2CH_2_), 52.86 (2C, 2CH), 131.64, 140.08, 152.16 (5C, Pyr-C), 167.33 (2C, 2C=O), 173.05 (2C, 2C=O), 168.08 (2C, C=N), 119.84, 129.35, 139.88, 150.35 (12C, Ar-C). MS (EI, 70 eV): *m/z* (%) = 716 [M^+^, 16], 537 (22), 389 (18), 276 (32), 206 (100). C_35_H_41_N_9_O_8_ (715.76): calcd. C 58.73, H 5.77, N 17.61; found C 58.65, H 5.72, N 17.55.

*Synthesis of 3,5-Bis-Hydrazone Derivatives* **10a**–**e***.* A mixture of the hydrazide derivative **4** (1 mmol) and the appropriate aldehydes, namely, benzaldehyde, *p*-methyl-, *p*-methoxy-, *p*-nitro-, and *p*-chlorobenzaldehydes (2 mmol) in absolute ethanol (50 mL) was heated under reflux for 6 h. The solvent was evaporated under reduced pressure and the residue was solidified with ether. The solid was collected by filtration, washed with ether and crystallized from a proper solvent to afford the corresponding 3,5-bis-hydrazone derivatives **10a**–**e**, respectively. 

*2,6-Bis {N-{(2R)-1-[2-(benzylidene)hydrazinyl]-4-methyl-1-oxopentan]-3-carboxamido}-pyridine* (**10a**). Yield: 68%; m.p. 216–218 °C (dioxane); 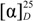: −145.0 (c = 0.5, DMF). IR (KBr): *ν* = 3510–3447 (NH), 3038 (CH-Ar), 2955 (CH-aliph.), 1656, 1528, 1319 (C=O, amide I, II and III) cm^−1^. ^1^H-NMR: *δ* = 1.04–0.95 (m, 12H, 4 CH_3_), 1.86–1.70 (m, 6H, 2 CH_2_ + 2CH), 4.65 (t, 2H, 2CHNH), 5.60 (s, 2H, 2CH=N), 7.45–7.72 (m, 10H, Ar-H), 8.42, 9.10 (2s, 4H, 4NH, exchangeable with D_2_O), 8.35, 9.18 (2s, 3H, Pyr-H). ^13^C-NMR: *δ* = 22.76 (4C, 4CH_3_), 23.01 (2C, 2CH), 41.16 (2C, 2CH_2_), 52.86 (2C, 2CH), 131.48, 140.32, 151.98 (5C, Pyr-C), 167.18 (2C, 2C=O), 173.05 (2C, 2C=O), 143.05 (2C, C=N), 128.68, 129.08, 130.85, 135.75 (12C, Ar-C). MS (EI, 70 eV): *m/z* (%) = 598 [M^+^, 24], 520 (15), 478 (18), 444 (10), 360 (100), 247 (32). C_33_H_39_N_7_O_4_ (597.71): calcd. C 66.31, H 6.58, N 16.40; found C 66.24, H 6.50, N 16.32.

*2,6-Bis {N-{(2R)-1-[2-(4-methylbenzylidene)hydrazinyl]-4-methyl-1-oxopentan]-3-carboxamido}-pyridine* (**10b**). Yield: 72%; m.p. 246–248 °C (AcOH); 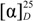: −130.5 (c = 0.5, DMF). IR (KBr): *ν* = 3488–3432 (NH), 3065 (CH-Ar), 2952 (CH-aliph.), 1652, 1533, 1322 (C=O, amide I, II and III) cm^−1^. ^1^H-NMR: *δ* = 1.00–0.95 (m, 12H, 4 CH_3_), 1.78–1.72 (m, 6H, 2 CH_2_ + 2CH), 2.24 (s, 6H, 2CH_3_), 4.68 (t, 2H, 2CHNH), 5.58 (s, 2H, 2CH=N), 6.98–7.56 (m, 8H, Ar-H), 8.38, 9.12 (2s, 4H, 4NH, exchangeable with D_2_O), 8.26, 9.05 (2s, 3H, Pyr-H). ^13^C-NMR: *δ* = 22.76 (4C, 4CH_3_), 22.95 (2C, 2CH), 24.12 (2C, 2CH_3_), 41.42 (2C, 2CH_2_), 52.76 (2C, 2CH), 131.46, 139.98, 152.15 (5C, Pyr-C), 166.95 (2C, 2C=O), 173.02 (2C, 2C=O), 142.55 (2C, C=N), 128.76, 129.01, 130.55, 139.85 (12C, Ar-C). MS (EI, 70 eV): *m/z* (%) = 626 [M^+^, 8], 534 (24), 402 (32), 360 (100), 246 (82). C_35_H_43_N_7_O_4_ (625.76): calcd. C 67.18, H 6.93, N 15.67; found C 67.09, H 6.84, N 15.60.

*2,6-Bis {N-{(2R)-1-[2-(4-methoxybenzylidene)hydrazinyl]-4-methyl-1-oxopentan]-3-carboxamido}-pyridine* (**10c**). Yield: 75%; m.p. 217–219 °C (EtOH); 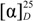: −98.5 (c = 0.5, DMF). IR (KBr): *ν* = 3490–3412 (NH), 3056 (CH-Ar), 2975 (CH-aliph.), 1657, 1532, 1336 (C=O, amide I, II and III) cm^−1^. ^1^H-NMR: *δ* = 1.10–0.94 (m, 12H, 4 CH_3_), 1.83–1.75 (m, 6H, 2 CH_2_ + 2CH), 3.68 (s, 6H, 2OCH_3_), 4.62 (t, 2H, 2CHNH), 5.62 (s, 2H, 2CH=N), 7.08–7.42 (m, 8H, Ar-H), 8.36, 9.08 (2s, 4H, 4NH, exchangeable with D_2_O), 8.24, 9.24 (2s, 3H, Pyr-H). ^13^C-NMR: *δ* = 22.68 (4C, 4CH_3_), 23.00 (2C, 2CH), 41.32 (2C, 2CH_2_), 52.88 (2C, 2CH), 55.75 (2C, 2OCH_3_), 131.42, 140.05, 152.14 (5C, Pyr-C), 167.08 (2C, 2C=O), 172.85 (2C, 2C=O), 143.15 (2C, C=N), 113.95, 125.78, 130.02, 162.45 (12C, Ar-C). MS (EI, 70 eV): *m/z* (%) = 658 [M^+^, 6], 626 (12), 596 (15), 478 (56), 247 (100). C_35_H_43_N_7_O_6_ (657.76): calcd. C 63.91, H 6.59, N 14.91; found C 63.81, H 6.50, N 14.84.

*2,6-Bis {N-{(2R)-1-[2-(4-nitrobenzylidene)hydrazinyl]-4-methyl-1-oxopentan]-3-carboxamido}-pyridine* (**10d**). Yield: 65%; m.p. 256–258 °C (AcOH/H_2_O); 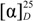: −142.5 (c = 0.5, DMF). IR (KBr): *ν* = 3492–3398 (NH), 3045 (CH-Ar), 2965 (CH-aliph.), 1657, 1532, 1331 (C=O, amide I, II and III) cm^−1^. ^1^H-NMR: *δ* = 1.01–0.95 (m, 12H, 4 CH_3_), 1.85–1.64 (m, 6H, 2 CH_2_ + 2CH), 4.62 (t, 2H, 2CHNH), 5.65 (s, 2H, 2CH=N), 7.68–8.00 (m, 8H, Ar-H), 9.10, 8.36 (2s, 4H, 4NH, exchangeable with D_2_O), 8.56, 9.10 (2s, 3H, Pyr-H). ^13^C-NMR: *δ* = 22.62 (4C, 4CH_3_), 22.95 (2C, 2CH), 41.40 (2C, 2CH_2_), 52.95 (2C, 2CH), 131.44, 140.10, 152.32 (5C, Pyr-C), 167.24 (2C, 2C=O), 172.88 (2C, 2C=O), 142.65 (2C, C=N), 120.05, 129.78, 139.65, 150.48 (12C, Ar-C). MS (EI, 70 eV): *m/z* (%) = 688 [M^+^, 16], 537 (22), 389 (18), 276 (32), 206 (100). C_33_H_37_N_9_O_8 _(687.70): calcd. C 57.63, H 5.42, N 18.33; found C 57.55, H 5.34, N 18.24.

*2,6-Bis {N-{(2R)-1-[2-(4-chlorobenzylidene)hydrazinyl]-4-methyl-1-oxopentan]-3-carboxamido}-pyridine* (**10e**). Yield: 62%; m.p. 225–227 °C (dioxane); 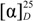: −116.45 (c = 0.5, DMF). IR (KBr): *ν* = 3490–3444 (NH), 3065 (CH-Ar), 2958 (CH-aliph.), 1656, 1522, 1318 (C=O, amide I, II and III) cm^−1^. ^1^H-NMR: *δ* = 1.10–0.95 (m, 12H, 4 CH_3_), 1.80–1.70 (m, 6H, 2 CH_2_ + 2CH), 4.68 (t, 2H, 2CHNH), 5.48 (s, 2H, 2CH=N), 7.26–7.68 (m, 8H, Ar-H), 9.08, 8.35 (2s, 4H, 4NH, exchangeable with D_2_O), 8.45, 9.36 (2s, 3H, Pyr-H). ^13^C-NMR: *δ* = 22.45 (4C, 4CH_3_), 22.90 (2C, 2CH), 41.33 (2C, 2CH_2_), 52.76 (2C, 2CH), 131.58, 140.22, 152.16 (5C, Pyr-C), 167.36 (2C, 2C=O), 172.91 (2C, 2C=O), 142.65 (2C, C=N), 128.50, 129.72, 131.94, 135.68 (12C, Ar-C). MS (EI, 70 eV): *m/z* (%) = 666 [M^+^, 6], 595 (12), 478 (45), 332(92), 260 (100). C_33_H_37_Cl_2_N_7_O_4_ (666.60): calcd. C 59.46, H 5.59, Cl 10.64, N 14.71; found C 59.40, H 5.53, Cl 10.60, N 14.65.

*Synthesis of Macrocyclic Octa-Bridged Peptide Derivatives* **11** *and* **12**. A suspension of **4** (0.34 g, 1 mmol) and 1,2,4,5-benzenetetracarboxylic acid dianhydride or 1,4,5,8-naphthalenetetracarboxylic acid dianhydride (1 mmol) in acetic acid (50 mL) was refluxed for 7 h. The obtained solid was collected by filtration, and crystallized from DMF/EtOH to give the corresponding macrocyclic candidates **11** and **12**, respectively.

*Macrocyclic* (**11**): Yield: 75%; m.p. 296–298 °C; 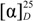: −112.6 (c = 0.5, DMF). IR (KBr): *ν* = 3478–3575 (NH), 3060 (CH-Ar), 2966 (CH-aliph.), 1753 (C=O, imide), 1656, 1530, 1320 (C=O, amide I, II and III) cm^−1^. ^1^H-NMR: *δ* = 0.98–0.90 (m, 24H, 8 CH_3_), 1.80–1.72 (m, 12H, 4 CH_2_ + 4 CH), 4.55 (t, 4H, 4CHNH), 9.08 (s, 4H, Ar-H), 8.78, 9.15 (2s, 8H, 8NH, exchangeable with D_2_O), 8.40, 9.20 (2s, 6H, Pyr-H). ^13^C-NMR: *δ* = 22.42 (8C, 8CH_3_), 22.98 (4C, 4CH), 41.18 (4C, 4CH_2_), 53.15 (4C, 4CH), 131.55, 140.08, 152.24 (10C, 2 Pyr-C), 167.16 (4C, 4C=O), 172.84 (4C, 4C=O), 164.16 (8C, 8C=O), 124.98, 134.85 (12C, Ar-C). MS (EI, 70 eV): *m/z* (%) = 1207 [M^+^, 4], 737 (5), 470 (32), 300 (100), 274 (72), 247 (85). C_58_H_58_N_14_O_16_ (1207.17): calcd. C 57.71, H 4.84, N 16.24; found C 57.65, H 4.78, N 16.20.

*Macrocyclic* (**12**). Yield: 56%; m.p. > 300 °C; 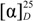: −124.5 (c = 0.5, DMF). IR (KBr): *ν* = 3544–3432 (NH), 3050 (CH-Ar), 2964 (CH-aliph.), 1742 (C=O, imide), 1657, 1530, 1326 (C=O, amide I, II and III) cm^−1^. ^1^H-NMR: *δ* = 1.05–0.98 (m, 24H, 8 CH_3_), 1.82–1.75 (m, 12H, 4 CH_2_ + 4 CH), 4.58 (t, 4H, 4 CHNH), 8.15–8.25 (m, 8H, Ar-H), 8.56, 9.16 (2s, 8H, 8NH, exchangeable with D_2_O), 8.40, 9.04 (2s, 6H, 2 Pyr-H). ^13^C-NMR: *δ* = 22.64 (8C, 8CH_3_), 22.95 (4C, 4CH), 41.35 (4C, 4CH_2_), 52.78 (4C, 4CH), 131.54, 140.12, 152.18 (10C, 2 Pyr-C), 167.36 (4C, 4C=O), 170.05 (4C, 4C=O), 158.68 (8C, 8 C=O), 120.25, 135.10, 139.73 (20C, Ar-C). MS (EI, 70 eV): *m/z* (%) = 1307 [M^+^, 4], 1014 (8), 787 (12), 654 (24), 294 (100), 246 (85). C_66_H_62_N_14_O_16_ (1307.28): calcd. C 60.64, H 4.78, N 15.00; found C 60.55, H 4.70, N 14.95.

### 3.2. Biological Activities

#### 3.2.1. Antimicrobial Activity

The antimicrobial activities of the synthesized compounds were determined by the agar diffusion method as recommended by the national committee for clinical laboratory standards (NCCLS) [28]. The concentrations of the tested compounds (10 µg/mL) were used according to modified Kirby–Bauer’s disk diffusion method [29]. The degree of inhibition is measured in comparison with that of chloramphinicol and fusidic acid taken as standards. 

#### 3.2.2. Anti-Inflammatory Activity

##### 3.2.2.1. Carrageenan-Induced Edema (Rat Paw Test)

Groups of adult male albino rats (150–180 g), each of eight animals were orally dosed with tested compounds at a dose level of 2.5–5 mg/kg one hour before carrageenan challenge. Foot paw edema was induced by subplantar injection of 0.05 cm^3^ of a 1% suspension of carrageenan in saline into the plantar tissue of one hind paw. An equal volume of saline was injected to the other hand paw and served as control. Four hours after drug administration the animals were decapitated, blood was collected, and the paws were rapidly excised. The average weight of edema was examined for the treated as well as the control group and the percentage inhibition of weight of edema was also evaluated. Diclofenac potassium (5 mg/kg) was employed as standard reference against which the tested compounds were compared (Table 2).

##### 3.2.2.2. *Estimation of Plasma Prostaglandin E2 (PGE2)*

Heparinized blood samples were collected from rats (n = 8), plasma was separated by centrifugation at 12,000 g for 2 min at 40 °C, immediately frozen, and stored at 20 °C until use. The design correlate EIA prostaglandin E2 (PGE2) kit (Aldrich, Steinheim, Germany) is a competitive immuno assay for the quantitative determination of PGE2 in biological fluids. The kit uses a monoclonal antibody to PGE2 to bind, in a competitive manner, the PGE2 in the sample after a simultaneous incubation at room temperature. The excess reagents were washed away and the substrate was added, after a short incubation time the enzyme reaction was stopped, and the yellow color generated was read on a microplate reader DYNATech, MR 5000 at 405 nm (Dynatech Industries Inc., McLean, VA, USA). The intensity of the bound yellow color is inversely proportional to the concentration of PGE2 in either standard or samples.

### 3.3. Anticancer Activity

#### 3.3.1. *In Vitro* Anti-Cancer Activities

The cytotoxicity of the newly synthesized compounds against cancer cell lines *in vitro* was performed with the MTT assay according to the Mosmann’s method. The MTT assay is based on the reduction of the soluble 3-(4,5-methyl-2-thiazolyl)-2,5-diphenyl-2*H*-tetrazolium bromide (MTT) into a blue-purple formazan product, mainly by mitochondrial reductase activity inside living cells. The cells used in cytotoxicity assay were cultured in RPMI 1640 medium supplemented with 10% fetal calf serum. Cells suspended in the medium (2 × 10^4^ mL^−^^1^) were plated in 96-well culture plates and incubated at 37 °C in a 5% CO_2_ incubator. After 12 h, the test sample (2 μL) was added to the cells (2Ý 104) in 96-well plates and cultured at 37 °C for 3 days. The cultured cells were mixed with 20 μL of MTT solution and incubated for 4 h at 37 °C. The supernatant was carefully removed from each well and 100 μL of DMSO were added to each well to dissolve the formazan crystals which were formed by the cellular reduction of MTT. After mixing with a mechanical plate mixer, the absorbance of each well was measured by a microplate reader using a test wavelength of 570 nm. The results were expressed as the IC_50_, which inducing a 50% inhibition of cell growth of treated cells when compared to the growth of control cells. Each experiment was performed at least three times. There was a good reproducibility between replicate wells with standard errors below 10%.

#### 3.3.2. Human Breast Cancer Xenograft Models and Animal Treatment

The animal protocol was approved by the Institutional Animal Use and Care Committee of the University of Alabama at Birmingham. Female athymic pathogen-free nude mice (nu/nu, 4–6 weeks) were purchased from Frederick Cancer Research and Development Center (Frederick, MD). To establish MCF-7 human breast cancer xenografts, each of the female nude mice was first implanted with a 60-day sc slow release estrogen pellet (SE-121, 1.7 mg 17β-estradiol/pellet; Innovative Research of America, Sarasota, FL, USA). The next day, cultured MCF-7 cells were harvested from confluent monolayer cultures, washed twice with serum-free medium, resuspended and injected subcutaneously (s.c.) (5 × 10^6^ cells, total volume 0.2 mL) into the left inguinal area of the mice. For the MDA-MB-468 xenograft model, 5 × 10^6^ cells (total volume 0.2 mL) were injected s.c. into the left inguinal area of the mice. All animals were monitored for activity, physical condition, body weight, and tumor growth. Tumor size was determined by caliper measurement in two perpendicular diameters of the implant every other day. Tumor weight (in g) was calculated by the formula, 1/2*a* × *b*^2^ where “*a*” is the long diameter and “*b*” is the short diameter (in cm).

The animals bearing human cancer xenografts were randomly divided into various treatment groups and a control group (7–10 mice/group). The untreated control group received the vehicle only. For the MCF-7 xenograft model, each one the newly synthesized tested compounds was dissolved separatly in PEG400/ethanol/saline (57.1:14.3:28.6, v/v/v), and was administered by intraperitoneal (i.p.) injection at doses of 10 mg/kg/d, 3 d/wk for 3 weeks [30]. 

## 4. Conclusions

A series of linear and macrocyclic peptides **3**–**12** were synthesized using 3,5-pyridinedicarboxylic acid as starting material and screened for their antimicrobial, anti-inflammatory and anticancer activities. The newly synthesized compounds **3**–**12** were tested as antimicrobial agents against different microorganisms. Regarding the protection against carrageenan-induced edema, all tested compounds, were found to be more potent than diclofenac potassium For these compounds, a similar activity profile was realized for the inhibition of plasma PGE2. Regarding the antiinflammatory activities the descending order of activity is **6**, **12**, **10a**, **7a**, **4**, **3**, **5**, **10d**, **7c**, **9a**, **9b** and **8**. Compounds **6**, **12**, **10a**, **7a** and **4** are the most active products. The *in vivo* anti-tumor activity of the tested compounds was evaluated in an MCF-7 mouse xenograft model of breast cancer and the descending order of activity was as follows: **10a**, **5**, **4**, **3**, **10d**, **12**, **9b**, **6**, **7a**, **9a**, **7c**.
